# Long-Term Infliximab Treatment in Psoriasis Patients: A National Multicentre Retrospective Study

**DOI:** 10.1155/2020/2042636

**Published:** 2020-03-09

**Authors:** Clémentine Carlet, Damien Bichard, Marie Aleth Richard, Emmanuel Mahé, Clémence Saillard, Emilie Brenaut, Alain Dupuy, Laurent Misery, Axel Villani, Denis Jullien, Eve Puzenat, Charlée Nardin, François Aubin, Groupe de Recherche sur le Psoriasis de la Société Française de Dermatologie

**Affiliations:** ^1^Dermatology Dpt, CHU Besançon, Besançon, France; ^2^Pharmacology Dpt, CHU Besançon, Besançon, France; ^3^Dermatology Dpt, CHU Marseille, Marseille, France; ^4^Dermatology Dpt, CH Victor Dupouy, Argenteuil, France; ^5^Dermatology Dpt, CHU Rennes, Rennes, France; ^6^Dermatology Dpt, CHU Brest, Brest, France; ^7^Dermatology Dpt, Hôpital Edouard Herriot, Lyon, France; ^8^French Society of Dermatology, Paris, France

## Abstract

**Background:**

Although infliximab (IFX) has been available since 2005, there are very little data on the long-term drug survival of infliximab in real-life.

**Objective:**

Our aim was to identify and describe psoriasis patients treated with IFX for longer than 6 years.

**Methods:**

Psoriasis patients treated with IFX for longer than 6 years were retrospectively included. Demographic and clinical data were collected in May 2018.

**Results:**

Between January 2005 and December 2012, 43 patients were maintained on IFX for 6 years or longer. IFX was introduced as a 4.5 line of systemic therapy. The mean duration of IFX treatment was 8.5 years (6–12). In May 2018, 30 patients (70%) were still maintained on IFX at 4–6 mg/kg every 8–10 weeks with an efficiency of about 100%. IFX was stopped in 13 patients (30%) mainly for loss of efficacy in 6 patients (46%). Three patients developed solid cancer including bladder cancer, lung cancer, and prostate cancer. *Limitation*. Retrospective study.

**Conclusion:**

We report the efficacy and safety of IFX maintained for up to 12 years in psoriasis patients. The long-term use of IFX was associated with a high BMI confirming the critical role of weight-based dosing for this drug.

## 1. Introduction

Infliximab (IFX) has been shown to be efficacious in patients with psoriasis across a number of randomized controlled trials [1]. Although IFX has been available since 2005, there are very little data on the long-term drug survival of infliximab in real-life [2]. Most of drug survival studies for biologics in psoriasis are limited to 5 years [3–5].

In a recent study describing our 12-year experience with biologics in psoriasis patients (6), we observed a mean treatment duration with IFX of 21.1 ± 26.7 months. In addition, the drug survival was lower with IFX than with the other biologics.

Long-term data are particularly important for therapies used over extended periods of time for the treatment of chronic conditions such as psoriasis. The aim of this paper was to investigate long-term IFX-treated psoriasis patients.

## 2. Patients and Methods

We conducted a national multicentre retrospective observational study asking members of the *Groupe de Recherche sur le Psoriasis of the Société Française de Dermatologie* (http://grpso.org) to report patients with psoriasis treated with IFX for 6 years or longer. Demographic data including, age, gender, body mass index (BMI), disease duration, comorbidities, and previous treatments were analyzed. Adverse events (AEs) were collected. As a real-life study, the decision of IFX maintenance was based on a clinical judgement of its efficacy rather than objective measurement of disease activity. The clinical judgement of efficacy is mainly based on dermatologists' knowledge and experience and patients' judgement of efficacy and tolerance.

## 3. Results

The prevalence of psoriasis patients treated with IFX for longer than 6 years was calculated in 3 centres (Argenteuil, Besançon, and Marseille) based on the total number of registered psoriasis patients with IFX. This number was not known in the other centres. Between January 2005 and December 2012, IFX was initiated in 209 patients, and 29 patients (14%) were treated for longer than 6 years. In addition, when considering all 6 participating centres, 43 patients (30 males and 13 women) were maintained on IFX for 6 years or longer between January 2005 and December 2012. The mean duration of psoriasis was 23 years (3–58). The mean age at IFX introduction was 58.7 (32–83), and IFX was introduced as a late line of systemic therapy (mean 4.5 lines). The mean duration of IFX treatment was 8.5 years (6–12). In terms of comorbidities, dyslipidemia was observed in 15 patients (35%), and depressive disorders were treated in 13 patients (30%). Body mass index (BMI) > 25 kg/m^2^ was observed in 31 patients (72%) and BMI was >30 kg/m^2^ in 13 patients (30%).

In May 2018, 30 patients (70%) were still on IFX at 4–6 mg/kg every 8–10 weeks with an efficiency of 100%. IFX dosing was increased to 6 and 10 mg/kg in 5 patients and methotrexate was added in 1 patient. IFX was stopped in 13 patients (30%) mainly for loss of efficacy in 6 patients. Three patients developed solid cancer including bladder, lung, and prostate cancers (after 6, 8, and 9 years, respectively). Based on patients' wishes, 2 patients were switched to other subcutaneous biologics. One patient developed an infusion reaction after 6 yr and one patient was lost to follow-up.

## 4. Discussion

Although the prevalence of long-term IFX-treated patients is not known, previous studies demonstrated a drug survival of IFX inferior to 20% at 6 years [4, 6]. In our series, we calculated a prevalence of 14% of patients who were treated with IFX for longer than 6 years. In our study, the mean duration of IFX treatment was 8.5 years.

Interestingly in these patients, the efficacy of IFX was maintained over time, since at the end of our study, 70% of patients were still maintained on IFX.

The long-term use of IFX was associated with a high prevalence of dyslipidemia (35%) and overweight (72%) including obesity in 30% of patients. Although IFX is associated with weight gain in patients with psoriasis [5], our study cannot distinguish the effect of IFX on the weight regulation. It was previously reported that besides the type of biologic agent, the statistically significant predictors of drug persistence were a low BMI [5, 7, 8] and the association with PsA or inflammatory bowel diseases [4, 7, 9], whatever the drug used. In a previous study [10], we retrospectively analyzed factors associated with long-term drug survival of infliximab in psoriasis patients. The mean duration of IFX survival was 41 months and the percentage of patients with a BMI <30 was not significantly different as compared to patients who experienced secondary loss of response within a 12-month period. Our long-term data suggest the critical role of weight-based dosing for IFX which may be considered as the ideal TNF blocker to treat obese patients [11].

The high prevalence of depressive disorders (30%) in our series suggests the impact of IFX on psychiatric illness [12]. Accumulating evidence implicates inflammation as an important contributor to the pathophysiology of depression and presents the immune system as a viable therapeutic target. Although ongoing clinical trials aim to investigate the effects of anti-TNF-alpha biologic infliximab on measures of anhedonia [13], in a recent 12-week, randomized, double-blind, placebo-controlled trial, IFX did not significantly reduce depressive symptoms compared with placebo in adults with bipolar depression [14].

In our long-term IFX-treated patients, the main reason for IFX discontinuation was the loss of efficacy (46%) as previously observed in patients treated for shorter periods [3, 4, 7, 15]. The second major reason was the delayed occurrence of a solid cancer in 3 patients (7%). The evidence to date suggests that there is no increased risk of cancers other than nonmelanoma skin cancer [16]. However, there is no “real-world” evidence and there are significant limitations to the studies identified, with the data largely from relatively short-term randomized controlled trials and open-label extension studies, making it difficult to extrapolate to real-world practice. In a real-world analysis of 16,545 biologic-naïve patients, Sbidian et al. [4] found a prevalence of solid cancer in 2.9% of IFX-treated patients with a 3.6 years mean follow-up. In a practical real-life 12-year experience including 77 psoriasis patients treated with IFX [6], 2.6% patients developed solid cancer after a mean follow-up of 4.1 years. Although it was difficult to attribute these cancers to the long-term use of IFX, our data suggest that long-term pharmacovigilance is still required.

In conclusion, our study demonstrates the long term sustained benefit of IFX in a small proportion of psoriasis patients, particularly in obese patients. However, there remains a need for ongoing pharmacovigilance in relation to cancer risk or the delayed onset of cancer and long-term use of biological therapy.

## Data Availability

The data used to support the findings of this study are included within the supplementary information file. The data used to support the findings of this study are restricted by the Besançon University Hospital in order to protect patient privacy. Data are available from Prof. François Aubin, Department of Dermatology, CHU, Besançon, France, for researchers who meet the criteria for access to confidential data. The data used to support the findings of this study are available from the corresponding author upon request.

## Supplementary Materials

Supplementary Materials Table 1: demographic data of patients maintained on IFX for longer than 6 years.
