# Supplementary material for: Long-Term Infliximab Treatment in Psoriasis Patients: A National Multicentre Retrospective Study
Source: Dermatol Res Pract. 2020 Mar 9;2020:2042636. doi: 10.1155/2020/2042636 (PMC7085387; doi:10.1155/2020/2042636)
Supplement: Supplementary Materials — Table 1: demographic data of patients maintained on IFX for longer than 6 years. [file 2042636.f1.doc]

**Supplementary File**

**Table 1 : Demographic data of patients maintained on IFX for longer than 6 years.**

BCC: basal cell carcinoma; CD : Crohn’s disease ; HS: hidradenitis suppurativa; HT : arterial hypertension ; IFX*: infliximab (5 mg/kg) every 8 weeks; L: line of treatment; LOE: loss of efficacy; PsoA : psoriatic arthritis ; SECU: secukinumab; SCC: squamous cell carcinoma; UST: ustekinumab;

Y: year.

| **Patient/Age (yrs)**  **Year of IFX introduction** | **BMI** | **IFX line (L)**  **Co-morbidities** | **Adverse Event** | **Current treatment** |
| --- | --- | --- | --- | --- |
| 1/66  2006 | 31.4 | L*4,  CD*,  Dyslipemia,  Depressive disorder | BCC* at Y10 | 2017 : switch to UST* |
| 2/72  2009 | 28.6 | L7,  Diabetes,  Dyslipemia,  Cardiovascular | Orchiepididymitis at Y*1  Coronary stent at Y6 | 2016 : switch to SECU* |
| 3/51  2007 | 28.7 | L5  Dyslipemia  Depressive disorder |  | IFX* |
| 4/45  2009 | 21.5 | L4  Spondylarthropathy  Uveitis |  | IFX |
| 5/37  2009 | 23.5 | L7 | Scabies at Y1  Folliculitis at Y2 | 2010 : IFX at 6 mg/kg |
| 6/42  2011 | 34 | L3  Dyslipemia  PsoA  Sleep apnea | PsoA* flare at Y1  Erysipela at Y3  Urticaria at Y6 | 2017 : switch to UST |
| 7/60  2007 | 33.4 | L7  Pso A  Dyslipemia  HT* | Pneumonia  2013: bladder cancer | 2013 : switch to acitretin |
| 8/80  2010 | 32.2 | L4  HT  Depressive disorder | PsoA at Y3  Pulmonary embolism at Y6 and Y8 | IFX |
| 9/43  2011 | 31.9 | L5  Dyslipemia |  | 2013: IFX at 6 mg/Kg |
| 10/51  2008 | 29 | L6  Diabetes |  | IFX |
| 11/ 74  2008 | 29.3 | L6  HT | SCC* at Y2 and Y7  CBC at Y3 | IFX |
| 12/ 63  2008 | 25.4 | L5  HT  Chronic limb ischemia | Ocular infection at Y9 | IFX |
| 13/ 41  2008 | 18.4 | L5  Depressive disorder |  | 2015 : IFX at 10 mg/kg  2016: LOE and switch to ADA |
| 14/ 57  2005 | 28.4 | L5  HT  Dyslipemia  Chronic renal insufficiency  Pancreatitis | Myocardial infaction at Y5  Lung cancer at Y8 (2013) | 2013 : stop IFX |
| 15/53  2010 | 31.2 | L4 |  | 2015 : IFX at 4 mg/kg |
| 16/60  2010 | 18.2 | L3 |  | IFX |
| 17/72  2005 | 26.5 | L6  Depressive disorder |  | 2011 : LOE* and switch to ADA |
| 18/64  2010 | 37.6 | L4  Depressive disorder Alcohol intake |  | +UV, 10 mg/kg  2016 : LOE and switch to SECU |
| 19/50  2010 | 22.5 | L3  PsoA  Dyslipemia |  | IFX |
| 20/60  2010 | 23.8 | L1  PsoA |  | IFX |
| 21/68  2007 | 24.4 | L3  Dyslipemia | AIT 2017 | IFX |
| 22/51  2009 | 24.7 | L5  PsoA  HT  Depressive disorder |  | IFX |
| 23/83  2008 | 30.2 | L5 |  | IFX |
| 24/69  2007 | 24.1 | L4  PsoA  Depressive disorder |  | IFX |
| 25/54  2008 | 34.7 | L2  Dyslipemia  Depressive disorder  CD |  | IFX |
| 26/76  2009 | 31.8 | L11  PsoA  HT | Inf urinaire | IFX |
| 27/70  2010 | 21.5 | L5 |  | IFX |
| 28/55  2010 | 27.1 | L2 |  | IFX + MTX (2A) |
| 29/45  2009 | 29.1 | L5  HT |  | IFX |
| 30/73  2011 | 18.5 | L5  Dyslipemia  Cardiovascular |  | IFX at 8 mg/kg |
| 31/69  2008 | 27.9 | L8  Diabete  Dyslipemia |  | IFX |
| 32/60  2007 | 31.5 | L3  Dyslipemia  HT | 2015 : coronary artery bypass  2015 : Lewis Sumner syndrome | 2017 : LOE and switch to UST |
| 33/38  2007 | 28.4 | L4 |  | 2015 : LOE and switch to UST |
| 34/56  2007 | 22.4 | L6 |  | 2015: LOE and switch to UST and than ADA |
| 35/74  2007 | 29.4 | L6  PsoA  Dyslipemia  HT | 2016: prostate cancer | 2016 : switch to apremilast |
| 36/45  2008 | 27.5 | L3  PsoA |  | IFX/10 weeks |
| 37/68  2011 | 27.7 | L3  Depressive disorder |  | IFX |
| 38/72  2006 | 36 | L5  Dyslipemia  HT |  | IFX |
| 39/51  2010 | 31.2 | L2  Depressive disorder |  | IFX |
| 40/53  2010 | 26.3 | L4  Depressive disorder |  | IFX/10 weeks |
| 41/56  2007 | 26.2 | L3 |  | IFX |
| 42/64  2008 | 25.4 | L5 |  | IFX |
| 43/42  2008 | 29.1 | L3  Depressive disorder  HS |  | 2012 and loss of follow-up |
